# Correction

**Published:** 1842-05

**Authors:** 


### CORRECTION.

In page 12 of the list of graduates of the Medical Institute of Louisville in our last number, for Andrew P. Price of Circleville, O., read Andrew B. Price, of Centreville, O.

